# Circulating BDNF in pediatric neurodevelopmental disorders: a meta-analysis of case–control differences and cognitive severity

**DOI:** 10.3389/fneur.2026.1958266

**Published:** 2026-09-23

**Authors:** Begüm Çapa Tayyare, Burcu Yuksel, Nurullah Eryılmaz

**Affiliations:** 1 Kocaeli Vocational School of Health Services, Kocaeli University, Kocaeli, Türkiye; 2 Institute of Educational Sciences, Leuphana University, Luneburg, Germany

**Keywords:** brain-derived neurotrophic factor, cerebral palsy, cognitive impairment, Down syndrome, intellectual disability, meta-analysis, neuroimmune biomarker

## Abstract

**Background:**

Brain-derived neurotrophic factor (BDNF) has been proposed as a neuroimmune biomarker of cognitive dysfunction in paediatric neurodevelopmental disorders other than autism spectrum disorder, but two related questions remain poorly characterised: whether circulating BDNF differs between affected and unaffected individuals, and whether it tracks the severity of cognitive impairment within affected populations. This systematic review and meta-analysis addressed both questions in children and adolescents with cerebral palsy (CP), intellectual disability (ID), or Down syndrome (DS).

**Methods:**

PubMed, Web of Science, Google Scholar, and citation-tracking (snowball) searches identified 340 records. After deduplication and title/abstract screening, 29 records underwent full-text assessment; 7 studies (comprising at least 547 participants) met eligibility criteria. Five comparisons (four studies, five case–control arms, 322 unique participants) reported extractable means and standard deviations and were pooled using Hedges’ *g* in a random-effects (DerSimonian-Laird) model; three additional studies, providing within-group severity-gradient data, were retained for narrative synthesis or sensitivity analysis because of missing healthy-control comparators, non-standard assay platforms, or data reported only in figures.

**Results:**

The case–control comparison yielded a pooled effect size of 0.46 (95% CI: −0.44 to 1.35), which was not statistically significant (*z* = 1.00, *p* = 0.32). Heterogeneity was very high (*Q* = 53.71, df = 4, *p* < 0.001; *I*^2^ = 92.6%; *τ*^2^ = 0.96). Subgrouping by diagnostic category did not reduce heterogeneity (*I*^2^ = 96.0% within studies of intellectual disability alone). A sensitivity analysis incorporating an additional digitised Down syndrome dataset yielded a consistent conclusion (pooled *g* = 0.54, 95% CI: −0.25 to 1.33).

**Conclusion:**

Current evidence does not support circulating BDNF as a reliable case–control or severity biomarker of cognitive impairment in children with CP, ID, or DS. The direction of association is inconsistent even within the same diagnostic category, likely reflecting methodological heterogeneity (assay platform, age-matching of controls) rather than a genuine, replicable biological signal. Age-matched studies using standardised assay protocols are needed before BDNF can be considered for clinical or research use in this population.

## 1 Introduction

Cognitive impairment shapes long-term functional outcomes across several of the most common pediatric neurodevelopmental disorders. In cerebral palsy (CP), the most prevalent childhood motor disability, population-based registries indicate that roughly 38–52% of affected individuals also meet criteria for intellectual disability (1). Cognitive difficulties in this group, particularly in executive function, processing speed, and visuospatial domains, tend to persist from childhood into adulthood rather than resolving with age (2). Non-syndromic intellectual disability (ID) and Down syndrome (DS) are defined largely by the severity of cognitive impairment (3, 4), yet the peripheral biological correlates of that severity remain incompletely characterised (5). A peripheral, minimally invasive biomarker that tracks cognitive status in these populations could support early risk stratification, monitoring response to rehabilitative or pharmacological intervention, and enriching clinical trial samples (6). This possibility has motivated a growing, if scattered, body of biomarker research over the past two decades.

Brain-derived neurotrophic factor (BDNF) is among the most extensively studied candidates for this role. It is a member of the neurotrophin family, signalling primarily through the high-affinity tropomyosin receptor kinase B (TrkB), and is densely expressed in the hippocampus, cerebral cortex, amygdala, and cerebellum (7). BDNF–TrkB signalling supports neuronal survival and differentiation during development, promotes dendritic and synaptic growth, and is mechanistically required for hippocampal long-term potentiation, the cellular process most closely linked to learning and memory consolidation. Because BDNF crosses the blood–brain barrier in a bidirectional, receptor-mediated manner, and because platelet and peripheral blood mononuclear cell stores of BDNF are thought to at least partially reflect central nervous system production, serum and plasma BDNF concentrations have been proposed as an accessible proxy for central neurotrophic activity, motivating their measurement in a wide range of neurodevelopmental and neuropsychiatric conditions.

A second, increasingly emphasised line of evidence situates BDNF within a neuroimmune rather than a purely neurotrophic framework. Pro-inflammatory cytokines such as tumour necrosis factor-alpha (TNF-α) and interleukin-6 (IL-6) modulate BDNF secretion from activated T cells, B cells, and monocytes, and BDNF in turn influences microglial and astrocytic activity, so that circulating BDNF concentrations reflect the joint output of neurotrophic and inflammatory signalling rather than either process in isolation (8). This dual relevance is reflected in the design of several studies included in the present review, which measured BDNF alongside oxidative stress markers, cytokines, or C-reactive protein rather than in isolation (8, 9), and motivates framing BDNF here explicitly as a neuroimmune, rather than purely neurotrophic, biomarker candidate.

The relationship between peripheral BDNF and cognitive outcome has already received formal meta-analytic attention in autism spectrum disorder (ASD), where a systematic review and meta-analysis of blood BDNF concentrations found modestly elevated levels relative to typically developing controls, alongside considerable between-study heterogeneity (10). Because ASD is already covered by an existing quantitative synthesis, the present review deliberately excludes it and instead focuses on three major paediatric neurodevelopmental categories for which no equivalent meta-analysis currently exists: cerebral palsy, non-syndromic intellectual disability, and Down syndrome. Individual primary studies in this field span more than.

two decades: from early reports of altered neurotrophin concentrations in archived neonatal blood from children later diagnosed with intellectual disability or cerebral palsy (11), through subsequent case–control studies using standard serum ELISA platforms in intellectual disability and autism (12), non-syndromic intellectual disability (13), and Down syndrome (8, 14), to more recent work linking BDNF to a broader panel of oxidative stress and inflammatory markers across severity strata of intellectual disability (9) and to biomarker commonalities between adult cerebral palsy and geriatric mild cognitive impairment (15).

Despite this two-decade accumulation of primary data, the direction and consistency of the BDNF-cognition relationship in these populations have never been formally synthesised. Individual studies report divergent findings: some describe higher BDNF concentrations in affected children relative to controls, others lower concentrations, and others no difference at all. They also differ substantially in assay platform (standard sandwich ELISA, immunoaffinity chromatography, Luminex bead-based assay, or semi-quantitative immunoblot densitometry), in whether concentrations are expressed in absolute units or after logarithmic transformation, and in whether age-matched healthy controls, an internal severity gradient, or a clinically distinct comparator group (e.g., an older-adult cohort) is used as the reference. This methodological heterogeneity, layered on top of genuine clinical heterogeneity across CP, ID, and DS, makes it difficult to draw a coherent conclusion from the primary literature alone, and is exactly the kind of situation a systematic quantitative synthesis is meant to address. A recent systematic review of cognition in adults with CP illustrates the resulting uncertainty directly: it rated the evidence linking BDNF (and C-reactive protein) to cognitive performance in that population as being of low certainty, citing exactly this combination of small samples, inconsistent methodology, and imprecision (2).

The present systematic review and meta-analysis therefore addressed two related but distinct questions and had four specific aims: (1) to identify and critically appraise all available studies reporting circulating BDNF concentrations in children or adolescents with cerebral palsy, non-syndromic intellectual disability, or Down syndrome (2) to quantitatively pool, where methodologically appropriate, the effect sizes from studies with an eligible case–control comparison, testing whether circulating BDNF differs between affected and unaffected individuals, using a standardised mean difference metric capable of reconciling the different assay platforms and units used across studies; (3) to separately synthesise the smaller body of within-group evidence testing whether circulating BDNF tracks the severity of cognitive impairment among affected individuals, without conflating this question with the case–control comparison; and (4) to formally characterise the degree and likely sources of heterogeneity across studies, including diagnostic category, assay platform, and age-matching of comparator groups, in order to determine whether the existing evidence base is sufficient to support BDNF’s candidacy as a neuroimmune biomarker of either case–control status or cognitive severity in this population, or whether it instead points primarily to gaps that future primary research should address.

## 2 Materials and methods

### 2.1 Protocol and registration

This systematic review was conducted and is reported in accordance with the PRISMA 2020 (Preferred Reporting Items for Systematic Reviews and Meta-Analyses) statement (16). An *a priori* protocol specifying the eligibility criteria, information sources, and the analysis.

plan was prepared before data extraction and is available from the authors. The protocol was finalized in June 2026, following a brief preliminary scoping search that indicated insufficient literature to support a review restricted to cerebral palsy alone; the population scope was broadened prior to protocol finalization to include non-syndromic intellectual disability and Down syndrome, as reflected in the eligibility criteria below. No deviations from the finalized protocol occurred during data extraction or analysis. The review was not registered in a public registry, which is acknowledged as a limitation.

### 2.2 Eligibility criteria

Inclusion criteria were: (1) Population children or adolescents diagnosed with cerebral palsy, non-syndromic intellectual disability, or Down syndrome (autism spectrum disorder was excluded *a priori* because it is already covered by an existing meta-analysis; see Section 1); (2) Exposure/index measure circulating BDNF concentration, measured in serum, plasma, or eluted from archived dried blood spots; (3) Comparator a healthy or typically developing control group where available, or an internal severity gradient (e.g., mild/moderate/severe) where no external control was reported; (4) Outcome a measure of cognitive function or intellectual disability severity; (5) Study design case–control, cohort, or cross-sectional; (6) English-language publication, with no restriction on publication year.

Exclusion criteria were populations defined by attention-deficit/hyperactivity disorder, specific learning disability, or autism spectrum disorder; studies of BDNF gene polymorphisms rather than circulating protein concentration; animal models; reviews or book chapters without original patient-level data; studies that did not measure BDNF; and duplicate reports of the same dataset (e.g., conference abstracts or posters superseded by a full-text article).

### 2.3 Information sources and search strategy

PubMed, Web of Science (Core Collection), and Google Scholar were searched; reference lists of included studies and relevant reviews were also screened (snowball/citation-tracking search). Institutional access to Scopus and the Cochrane Library was not available at the time of the search, in addition to the previously noted lack of Embase access. Search terms combined diagnostic-group terms (“cerebral palsy,” “intellectual disability,” “intellectual disability,” “Down syndrome”) with biomarker terms (“BDNF,” “brain-derived neurotrophic factor”) and cognitive-outcome terms (“cognitive impairment,” “IQ,” “intelligence”) using Boolean AND, with autism- and ADHD-related terms excluded via NOT. Access to Embase was not available through the authors’ institution and is noted as a limitation. Search date: from inception to June 2026.

### 2.4 Study selection and data extraction

Title/abstract screening and full-text assessment were performed independently by BCT, BY and NE, with disagreements resolved by discussion. For each included study, the following were extracted using a standardised form: diagnostic group, sample size, age range, BDNF assay method and unit, comparator group, cognitive/severity measure, and mean ± standard deviation (or correlation coefficient) for BDNF concentration. All extraction was carried out and checked by the three authors, with every entry verified against the source.

### 2.5 Statistical analysis

Because the included studies used heterogeneous BDNF units and assay platforms (pg./mL, ng/mL, log-transformed concentrations, and densitometric immunoblot intensity), Hedges’ *g* (bias-corrected standardised mean difference) was used as the common effect size metric (17). Positive values of Hedges’ *g* indicate higher BDNF concentrations in the affected (case) group relative to the comparator, and negative values indicate lower BDNF concentrations in the affected group. Pooling was performed using a random-effects model (DerSimonian-Laird method), reflecting the *a priori* expectation of methodological and population heterogeneity (18). Because Nelson et al. (11) reported two diagnostic-group comparisons (intellectual disability and cerebral palsy) against a single shared control group (*n* = 54), entering both comparisons at full control size would double-count the same control participants and artificially inflate their combined precision [unit-of-analysis error; (19)]. Following Cochrane Handbook guidance, the shared control group was therefore divided equally between the two comparisons (*n* = 27 per comparison) before computing Hedges’ *g* and its variance for each arm. Heterogeneity was quantified using Cochran’s *Q* and the *I*^2^ statistic (20). Given the small number of comparisons (*k* = 5) and very high heterogeneity, the random-effects model was additionally re-estimated using restricted maximum likelihood (REML) with the Hartung-Knapp-Sidik-Jonkman (HKSJ) adjustment to the standard error and confidence interval, which provides more conservative and better-calibrated inference under these conditions (28). A 95% prediction interval was also calculated to indicate the range of true effects expected in a future comparable study. Sensitivity analyses were conducted to assess the robustness of pooled estimates to (a) inclusion of a study whose data were digitised from a bar graph rather than reported numerically, and (b) restriction to a single broad diagnostic subgroup and (c) restriction to studies using conventional serum/plasma sampling versus archived dried blood spot eluates.

### 2.6 Risk of bias assessment

Risk of bias was assessed with QUIPS across its six domains: study participation, study attrition, prognostic factor measurement, outcome measurement, adjustment for other prognostic factors, and statistical analysis and reporting (27). Although QUIPS and the GRADE approach for prognostic factors were developed primarily for longitudinal prognostic studies, they were adapted here as the most structured available frameworks for appraising bias and certainty in a literature composed predominantly of cross-sectional and case–control designs; some domains (e.g., study attrition) are less directly applicable to this study type and were interpreted accordingly. All risk-of-bias coding was carried out and checked by the three authors, with domain and overall ratings agreed by consensus.

Domain and overall ratings are provided in Supplementary Table S1.

### 2.7 Certainty of the evidence (GRADE)

Certainty in the pooled effect estimate for circulating BDNF as a prognostic marker of cognitive impairment severity was assessed using the GRADE approach adapted for prognostic factor research (21), considering risk of bias, inconsistency, indirectness, imprecision, and publication bias. Ratings were assigned by consensus among the three authors and are presented in Table 1.

**Table 1:** GRADE assessment of certainty in the pooled effect estimate for circulating BDNF as a marker of cognitive impairment severity (main quantitative pool, *k* = 5 comparisons).

Domain	Rating	Rationale
Risk of bias	Serious ↓	Most studies contributing to the main quantitative pool [(11)—ID and CP arms; (12, 14)] were rated overall High risk of bias on QUIPS (Supplementary Table S1), driven principally by confounding and outcome-measurement concerns.
Inconsistency	Serious ↓	Very high heterogeneity (*I*^2^ = 92.6%, *τ*^2^ = 0.96); direction of effect reversed within the same diagnostic category [intellectual disability: (13) negative vs. (11) and (12) positive].
Indirectness	Serious ↓	Pooled estimate combines three distinct diagnostic populations (cerebral palsy, non-syndromic intellectual disability, Down syndrome) with distinct underlying etiologies under a single construct.
Imprecision	Serious ↓	Wide 95% confidence interval spanning the null (−0.44 to 1.35), derived from only five comparisons across four studies (*n* = 322); this imprecision is corroborated by the wider confidence interval obtained under REML/Hartung-Knapp estimation (−0.80 to 1.71) and a prediction interval spanning −2.95 to 3.87.
Publication bias	Not assessable	Number of studies too small (*k* = 5) for funnel-plot or Egger’s-test interpretation; cannot be ruled out.
Overall certainty	Very low	Four of five GRADE domains downgraded; the true effect may differ substantially from the pooled estimate reported here.

## 3 Results

### 3.1 Study selection

The search strategy identified 340 records in total [PubMed: 58; Web of Science: 78; Google Scholar: ~200 (first 200 results screened); another sources/snowball search: 4]. After removing duplicates, 280 records were screened at the title/abstract level, of which 250 were excluded as out of scope. The remaining 29 records underwent full-text review; 22 were excluded with documented reasons (wrong population, *n* = 8; wrong exposure type/genetic polymorphism, *n* = 3; animal model, *n* = 1; review without original data, *n* = 3; ineligible design/outcome, *n* = 4; duplicate dataset, *n* = 2; ineligible comparator group, *n* = 1). Seven studies were ultimately included in qualitative and/or quantitative synthesis (Figure 1, PRISMA flow diagram).

**Figure 1:**
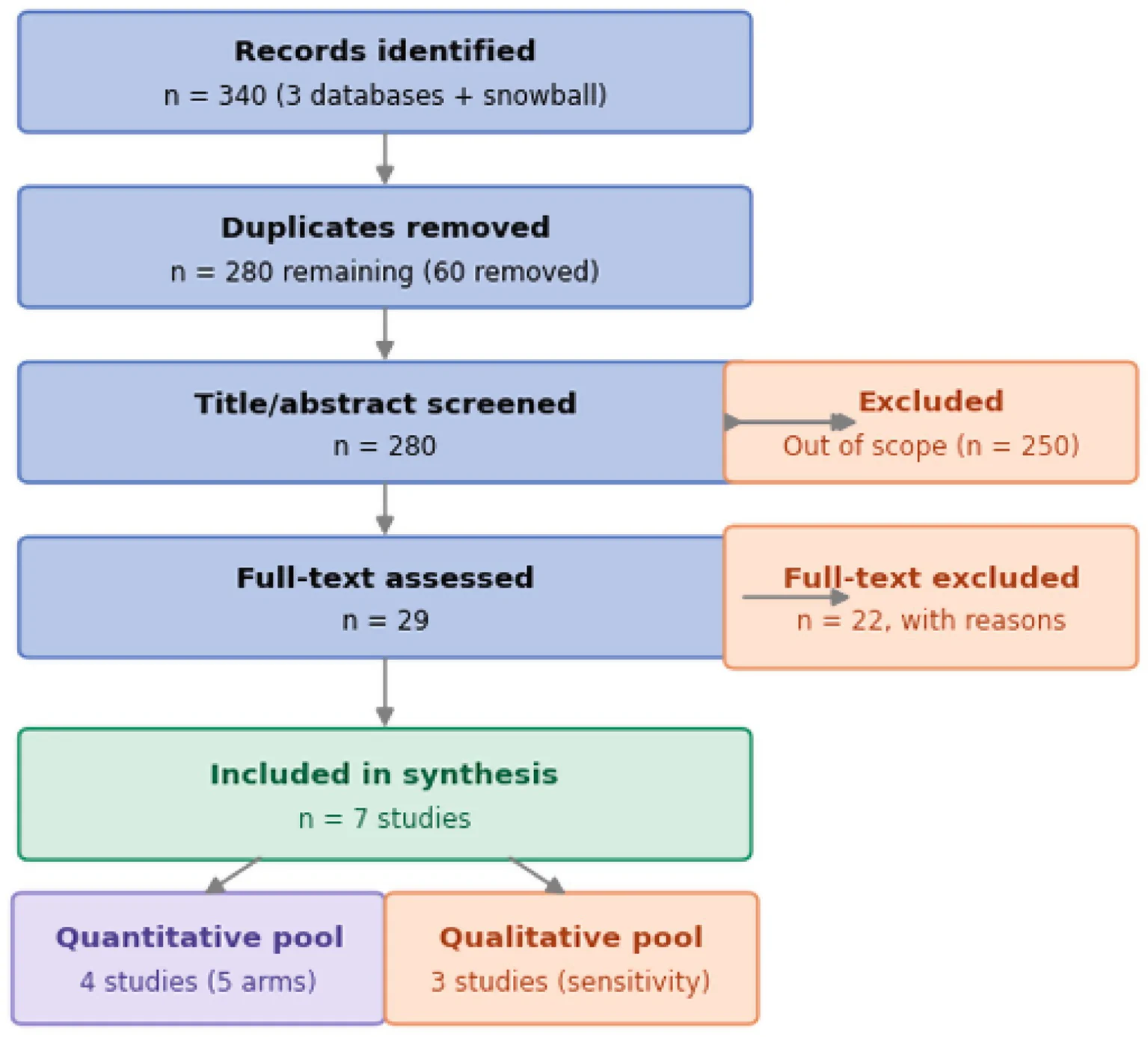
PRISMA flow diagram.

### 3.2 Characteristics of included studies

Table 2 summarises the characteristics of the seven included studies. Five comparisons (four studies, five case–control arms) reported means and standard deviations sufficient to compute Hedges’ *g* and were included in the main quantitative pool; three studies (8, 9, 22) were retained for sensitivity or qualitative synthesis owing to a missing external control group, uncertainty in data extraction, or a non-standard assay platform. Ng et al. (15), whose comparator group was an older-adult mild cognitive impairment (MCI) cohort rather than a healthy control or an internal severity gradient, did not meet the eligibility criteria specified in Section 2.2 and was therefore excluded from the review’s evidence base; it is discussed only as contextual related work in Section 4.

**Table 2:** Characteristics of the seven studies included in qualitative and/or quantitative synthesis.

Study	Population (*n* case/control)	Design	BDNF assay method	Finding (direction/significance)
Esnafoglu and Adıgüzel (13)	Non-syndromic ID (33/30)	Case–control	Serum ELISA	BDNF lower; positive correlation with IQ (*p* = 0.002)
Nelson et al. (11) (ID arm)	Intellectual disability (60/54)	Case–control, archived neonatal dried blood spot (eluate)	Immunoaffinity chromatography	BDNF higher (*p* < 0.0001)
Nelson et al. (11) (CP arm)	Cerebral palsy (63/54)	Case–control, archived neonatal dried blood spot (eluate)	Immunoaffinity chromatography	Not significant
Nelson et al. (14) (DS arm)	Down syndrome (26/20)	Case–control, archived neonatal dried blood spot (eluate)	Luminex + ELISA	Not significant
Miyazaki et al. (12)	Intellectual disability (20/16)	Case–control	Serum ELISA	BDNF higher (*p* < 0.001)
Tarani et al. (8)^*^	Down syndrome, prepubertal (9/21)	Case–control	Serum ELISA	BDNF higher (*p* = 0.04)
Gamayani et al. (22)^**^	Cerebral palsy, IQ severity strata (*n* = 150)	Cross-sectional (no external control)	Serum ELISA	Not significant (no severity gradient)
Sengupta et al. (9)	Intellectual disability, IQ severity strata (*n* = 45)	Cross-sectional (no external control)	Immunoblot (densitometric)	Not significant (after age adjustment)

^*^Tarani et al. (8): numerical values were not reported in text or table form and were digitized (approximated) from a bar graph in Figure 2 of the source article (DS: ~57 ± 16.5 pg/mL, *n* = 9; control: ~42 ± 13.7 pg/mL, *n* = 21); accordingly, this study was analyzed only in a sensitivity analysis, not the main pool.

^**^Gamayani et al. (22): sample size (*n* = 150) was confirmed by consulting the original source article (Int J Med Pharm Sci, 2015), which was not accessible through standard database indexing at the time of initial extraction.

The main quantitative pool comprised 322 unique participants [(13): *n* = 63; (11): *n* = 177, comprising 60 ID cases, 63 CP cases, and a single shared control group of 54 individuals used for both diagnostic arms; (14): *n* = 46; (12): *n* = 36]. Including the three additional studies retained for sensitivity or qualitative synthesis [(8): *n* = 30; (9): *n* = 45; (22): *n* =150], the seven included studies together contributed data from at least 547 participants.

### 3.3 Quantitative synthesis (meta-analysis)

Hedges' *g* values from five case-control comparisons [(11, 13), ID and CP arms; (14), DS arm; (12)] were pooled using a random-effects model. Because the two Nelson et al. (11) arms shared a single control group (see Section 2.5), that group's 54 individuals were divided equally (*n* = 27 per arm) before computing each arm's variance, avoiding double-counting the same participants' precision in the pooled estimate. The pooled effect size was 0.46 (95% CI: -0.44 to 1.35) and was not statistically significant (z = 1.00, p=0.32). Heterogeneity remained very high (*Q* = 53.71, df = 4, *p* < 0.001; *I*^2^=92.6%; *τ*^2^=0.96) (Figure 2, forest plot; Table 3). This unit-of-analysis correction left the pooled estimate, its significance, and the overall heterogeneity essentially unchanged relative to the uncorrected calculation, indicating that the review's conclusions are not an artifact of the original double-counting.

**Figure 2:**
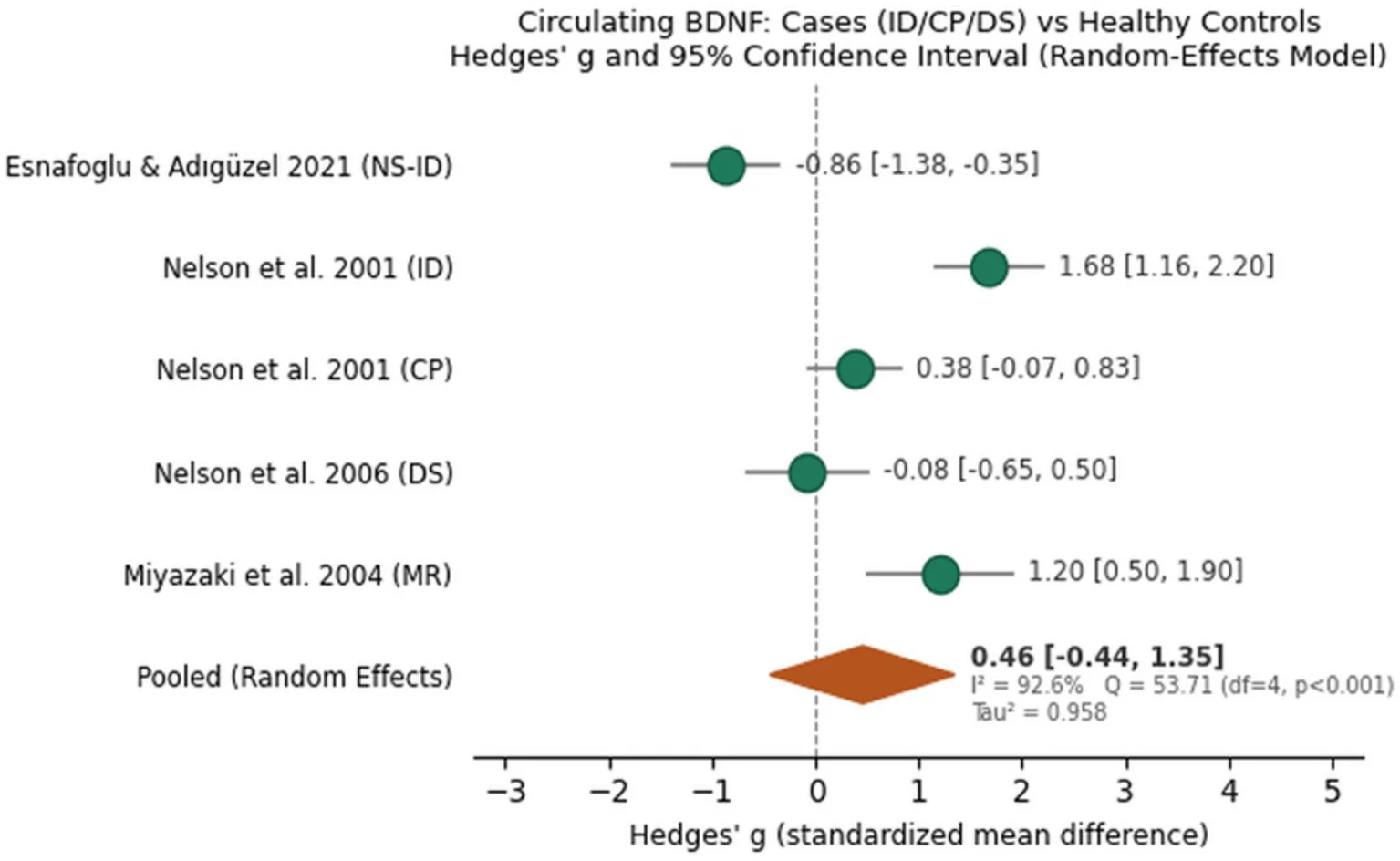
Circulating BDNF in cases (ID/CP/DS) versus healthy controls. Hedges' *g* and 95% confidence intervals, random-effects model.

**Table 3:** Effect sizes (Hedges' *g*) of the five case-control comparisons included in the main quantitative pool, random-effects model.

Study	Hedges’ *g*	95% CI	Weight (%)
Esnafoglu and Adıgüzel (13)	−0.86	−1.38, −0.35	20.2
Nelson et al. (11) (ID)	1.68	1.16, 2.20	20.2
Nelson et al. (11) (CP)	0.38	−0.07, 0.83	20.6
Nelson et al. (14) (DS)	−0.08	−0.65, 0.50	19.9
Miyazaki et al. (12)	1.20	0.50, 1.90	19.1
Pooled (random effects)	0.46	−0.44, 1.35	100

Under REML estimation with the Hartung-Knapp adjustment, the pooled estimate was materially unchanged (*g* = 0.46) but the 95% confidence interval was wider (−0.80 to 1.71, *p* = 0.37), reflecting the small number of comparisons (*k* = 5); see Section 3.3.

The direction of effect was inconsistent even within the same broad diagnostic category (intellectual disability): Esnafoglu and Adıgüzel (13) reported significantly lower BDNF, whereas Nelson et al. (11) and Miyazaki et al. (12) reported significantly higher BDNF in the same group. Re-estimating the model using REML with the Hartung-Knapp adjustment yielded a materially consistent point estimate (*g* = 0.46) but a wider, more conservative confidence interval (95% CI: -0.80 to 1.71, *p* = 0.37), reflecting the small number of comparisons (*k* = 5). The 95% prediction interval was −2.95 to 3.87, indicating that a future comparable study could plausibly find an effect in either direction of clinically meaningful magnitude.

### 3.4 Sensitivity and subgroup analyses

When the digitised Down syndrome dataset from Tarani et al. (8) was added to the main pool (six arms), the pooled effect was 0.54 (95% CI: −0.25 to 1.33), preserving both the lack of statistical significance and the high heterogeneity (*I*^2^ = 91.0%). This indicates that the overall conclusion is not sensitive to the digitisation uncertainty of this single dataset (Figure 3).

**Figure 3:**
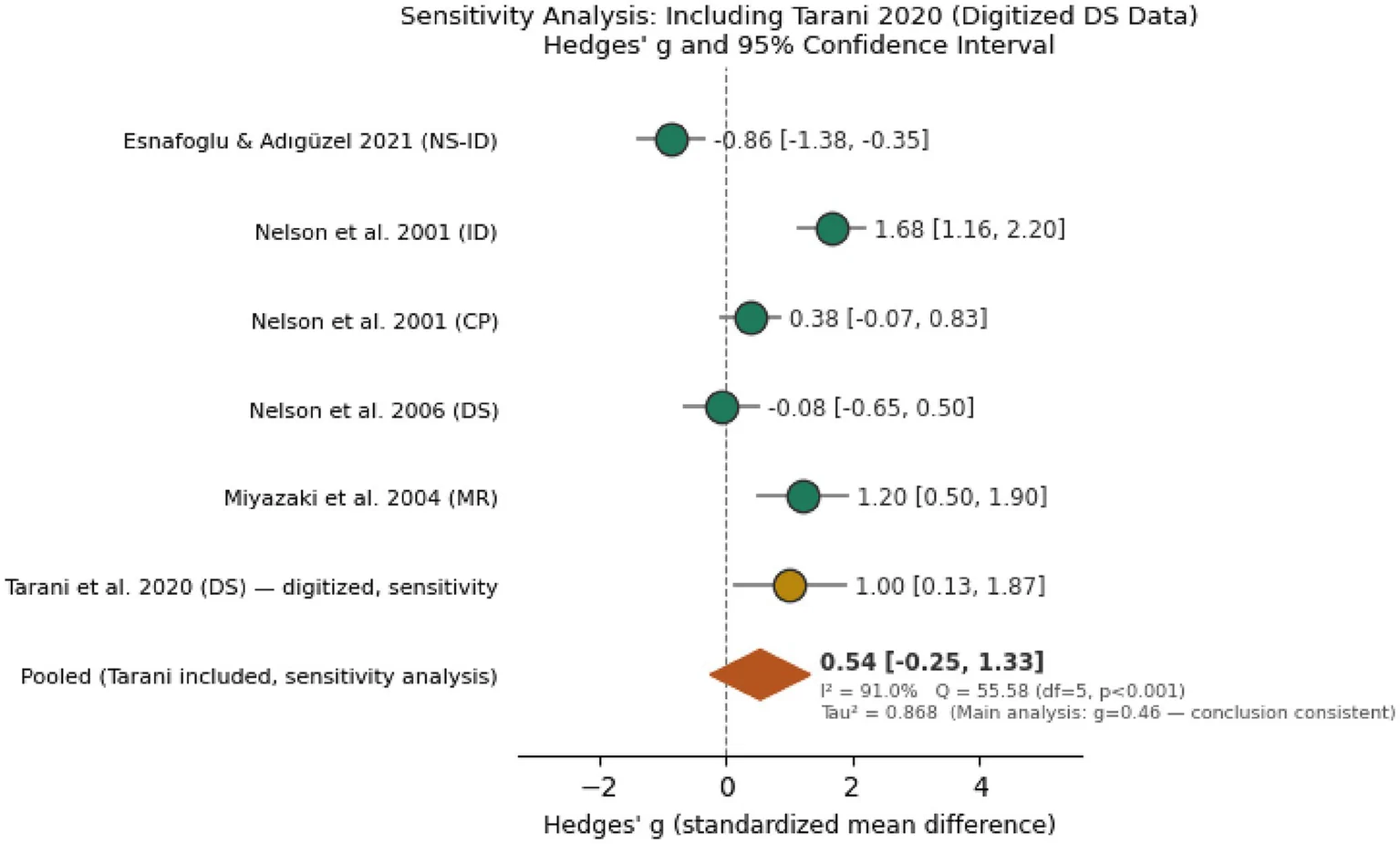
Sensitivity analysis: pooled effect size after including the digitized dataset of Tarani et al. (8).

A subgroup analysis restricted to studies of intellectual disability alone [(11, 13) ID arm (corrected control *n* = 27); (12); *n* = 3] yielded a pooled effect of 0.67 (95% CI: −0.99 to 2.33); heterogeneity was not reduced and in fact increased (*I*^2^ = 96.0%). This is consistent with the observed heterogeneity reflecting methodological factors such as assay platform or the age structure of the samples, at least in part, rather than diagnostic category alone; however, with only five comparisons in the main pool, this interpretation remains observational, as a formal moderator analysis (e.g., meta-regression) was not attempted given the well-recognized instability of such models at this sample size.

A specific methodological concern is that the control group in Miyazaki et al. (12) comprised adults (mean age 23.3 years), whereas the patient groups were children (mean ages 7.6–11.0 years). Nelson et al. (14) demonstrated that circulating BDNF concentration rises markedly with age (from a mean of 740 pg./mL in very-low-birth-weight preterm infants to 5,497 pg./mL in healthy adults). Accordingly, the “higher BDNF in patients” finding reported by Miyazaki et al. (12) should be interpreted with caution in the absence of age-matched controls.

Because Nelson et al. (11, 14) measured BDNF in eluates from archived neonatal dried blood spots rather than fresh serum or plasma, a further sensitivity analysis was restricted to the two studies using conventional serum sampling (12, 13). This yielded a pooled effect of g = 0.15 (95% CI: −1.86 to 2.17, *k* = 2, *I*^2^ = 95.2%), which remained non-significant with an even wider confidence interval, indicating that the null, imprecise overall conclusion is not an artifact of pooling across specimen types, though the very small number of studies in this subset limits the strength of this check.

### 3.5 Severity-gradient synthesis (within-group evidence)

The quantitative pool described above (Sections 3.3–3.4) addresses whether circulating BDNF differs between diagnosed and non-diagnosed groups (a case–control question), which is conceptually distinct from whether BDNF tracks the degree of cognitive impairment within an affected group (a severity-gradient question). Three included studies provided within-group severity data suitable for this second question; because they used incompatible effect-size metrics (a Pearson correlation, a one-way ANOVA across five ordinal severity strata, and an age-adjusted ANCOVA on a semi-quantitative densitometric measure), they could not be statistically pooled into a single summary effect and are instead synthesized narratively with their own quantitative anchors, summarized in Table 4.

**Table 4:** Severity-gradient (within-group) evidence for circulating BDNF and cognitive impairment severity; not statistically poolable across studies owing to incompatible effect-size metrics.

Study (design)	Effect/statistic	Interpretation
Esnafoglu and Adıgüzel (13) (case–control, IQ correlation)	*r* = 0.424, *p* = 0.001	Positive association, but conflates case–control and severity constructs
Gamayani et al. (22) (5-stratum severity gradient, *n* = 150, no control)	*F*(4,145) = 0.04, *η*^2^ = 0.001, *r* ≈ 0.03 (computed for this review)	Negligible; BDNF flat across severity strata
Sengupta et al. (9) (3-stratum severity gradient, *n* = 45, no control)	ANCOVA n.s. (age-adjusted); statistic not reported in source	No detectable severity gradient

Esnafoglu and Adıgüzel (13) reported a significant positive correlation between serum BDNF and performance IQ across their combined ID and control sample (*r* = 0.424, *p* = 0.001). Because this correlation was computed across a case–control sample rather than within a single diagnostic group stratified by severity, it reflects the case–control contrast at least in part rather than a pure within-group severity gradient.

Gamayani et al. (22), in the largest available within-group severity study (*n* = 150 children with CP stratified into five IQ-based severity strata, no external control), reported materially flat BDNF concentrations across strata (38.4–39.9 pg./mL; one-way ANOVA, *p* = 0.99). Using the study’s own reported group means, standard deviations, and sample sizes, we computed the corresponding effect size for this review: *F*(4,145) = 0.04, *η*^2^ = 0.001 (*r* ≈ 0.03), indicating a negligible association between BDNF and severity within this cohort.

Sengupta et al. (9), in a smaller within-group severity study (*n* = 45 children with intellectual disability, three strata), likewise reported that serum BDNF (measured by immunoblot densitometry) was unaltered across severity strata after adjustment for age (ANCOVA with Bonferroni correction); the original report did not provide a numerical effect size or test statistic for this comparison, so it is included here narratively rather than in Table 4.

Taken together, two of the three available within-group severity studies (9, 22)—together the largest severity-gradient samples in this literature—found no detectable relationship between circulating BDNF and cognitive severity, while the third (13) reported a significant correlation but in a case–control rather than a pure within-group severity design. This pattern indicates that even where BDNF differs between diagnosed and non-diagnosed groups (Sections 3.3–3.4), the existing evidence does not support BDNF as a marker that tracks the degree of impairment once a diagnosis is established, which directly limits its candidacy as a severity biomarker independent of its case–control performance.

### 3.6 Certainty of evidence and limitations

Applying the GRADE approach adapted for prognostic factor research (21), the certainty in the pooled effect estimate for circulating BDNF as a marker of cognitive impairment severity was rated as very low (Table 1). Certainty was downgraded for risk of bias, because most studies contributing to the main quantitative pool [(11) ID and CP arms; (12, 14)] were rated overall as high risk of bias on QUIPS (Supplementary Table S1), driven principally by confounding and outcome-measurement concerns; for inconsistency, given very high heterogeneity (*I*^2^ = 92.6%, *τ*^2^ = 0.96) and a reversal in the direction of effect within the same diagnostic category (intellectual disability); for indirectness, because the pooled estimate combines three distinct diagnostic populations (cerebral palsy, non-syndromic intellectual disability, Down syndrome) with distinct underlying etiologies under a single cognitive-impairment construct; and for imprecision, given a wide 95% confidence interval spanning the null (−0.44 to 1.35) derived from only five comparisons across four studies (*n* = 322). Publication bias could not be formally assessed because the number of studies was too small for funnel-plot or Egger’s-test interpretation (see below); the absence of a formal assessment should not be interpreted as evidence that publication bias is absent. This very low certainty rating means the true effect could differ substantially from the pooled estimate reported here, reinforcing the review’s overall conclusion that current evidence does not support circulating BDNF as a reliable biomarker of cognitive impairment severity in this population.

Most included studies were small and employed differing BDNF assay platforms (standard ELISA, immunoaffinity chromatography, and immunoblot densitometry). Institutional access to Embase was unavailable, which may have limited retrieval of additional eligible studies; this is a limitation of the present review. Data for Tarani et al. (8) were not originally reported in numerical form and were approximated by digitisation, introducing additional imprecision that is reflected in the corresponding sensitivity analysis.

## 4 Discussion

To our knowledge, this is the first systematic review and meta-analysis to quantitatively synthesise the relationship between circulating BDNF and the severity of cognitive impairment across cerebral palsy, non-syndromic intellectual disability, and Down syndrome. The principal finding is a pooled effect size that was imprecisely estimated rather than negligible (Hedges’ *g* = 0.46, 95% CI: −0.44 to 1.35; 95% CI: −0.80 to 1.71 under REML/Hartung-Knapp) that did not reach statistical significance, accompanied by very high heterogeneity (*I*^2^ = 92.6%). Together, these results indicate that the primary literature, as it currently stands, does not support a consistent, replicable association between circulating BDNF concentration and cognitive impairment in this population. Given how often BDNF is invoked as a candidate neurodevelopmental biomarker, this null result is notable.

The most striking feature of the pooled data is not the null point estimate but the underlying directionality problem. Esnafoglu and Adıgüzel (13) reported significantly lower serum BDNF in non-syndromic ID relative to typically developing controls, with a positive correlation between BDNF and IQ; by contrast, Nelson et al. (11) and Miyazaki et al. (12) reported significantly higher BDNF in children with intellectual disability relative to controls. These are not studies that merely failed to replicate one another’s null results; they report opposite directions of effect for ostensibly the same broad diagnostic construct. Restricting the meta-analysis to these three intellectual disability studies did not resolve this contradiction: subgroup-level heterogeneity was, if anything, higher (*I*^2^ = 96.0%) than in the full pooled analysis, indicating that diagnostic category is not the primary axis along which these studies differ.

Several methodological features plausibly explain this directional inconsistency better than diagnosis does. First, assay platforms differed substantially across studies: Esnafoglu and Adıgüzel (13) and Miyazaki et al. (12) used standard sandwich ELISA on fresh serum, whereas Nelson et al. (11, 14) measured BDNF in eluates from archived neonatal dried blood spots using single- or double-antibody immunoaffinity/Luminex platforms. The original investigators reported that these eluate-based assays were subject to substantial, non-linear interference from inhibitory substances, a technical limitation discussed in both Nelson et al. reports and not fully resolved between their 2001 and 2006 publications, which used different assay technologies (recycling immunoaffinity chromatography versus Luminex/ELISA) and did not agree on whether BDNF distinguished autism from controls. Second, and perhaps more consequentially, the age-matching of comparator groups varied widely. Miyazaki et al. (12) compared children with intellectual disability (mean age 7.6–11.0 years) against adult controls (mean age 23.3 years). Because Nelson et al. (14) demonstrated a marked, monotonic rise in circulating BDNF with age (from 740 pg./mL in very-low-birth-weight preterm infants, through 2,025 pg./mL in term newborns, to 5,497 pg./mL in healthy adults), an adult control group would, if anything, be expected to show higher, not lower, BDNF than paediatric patients on the basis of age alone. That Miyazaki et al. (12) nonetheless found higher BDNF in the younger patient groups makes their finding harder, not easier, to interpret in isolation, and is a reminder that age-matching should be a mandatory design feature in future BDNF studies in this population rather than an incidental covariate. This specimen-type distinction was formally tested in a dedicated sensitivity analysis (Section 3.4), which showed that restricting the pool to conventional serum/plasma studies did not materially change the overall conclusion, though it did widen an already imprecise estimate.

The cerebral palsy-specific evidence, on its own, is comparatively more consistent, though still limited. Nelson et al. (11) found that neonatal BDNF concentrations in children later diagnosed with CP did not differ from population-based controls, in contrast to the significant elevation observed in their intellectual disability and autism groups. Ng et al. (15), though not eligible for the present review (Section 2.2), found significantly lower BDNF in adults with CP relative to an MCI cohort, with no association to either of two neurocognitive measures within the CP group. Taken alongside Nelson et al. (11), this suggests, only as a hypothesis for future eligible research, that CP does not show the same BDNF elevation reported in some ID/mental-retardation samples, and that within CP, BDNF may be more informative about systemic/cardiometabolic aging [(15) proposed an “accelerated aging” model linking BDNF to Framingham cardiovascular risk scores and high-sensitivity CRP] than about cognitive status per se. This fits with a broader body of CP biomarker literature not eligible for the present quantitative pool but relevant to interpretation: Hansen et al. (23) linked reduced plasma BDNF in severe CP to suboptimal nutrition and low physical activity rather than to cognitive severity directly, and two small rehabilitation-intervention studies excluded from the main pool for lacking a healthy-control comparison reported opposite directions of BDNF change with improved motor/functional outcomes: an increase following 6 months of multidisciplinary rehabilitation in one cohort (24), and a decrease following a brain-computer-interface exoskeleton intervention in another (25). This contradiction may itself be informative, suggesting that BDNF is state-dependent and plastic in CP, responsive to intervention, systemic health, and possibly measurement timing, in ways a single cross-sectional case–control comparison cannot capture. This interpretation is consistent with preclinical evidence that systemic and neurological stressors can substantially modify hippocampal BDNF expression independent of any cognitive or developmental diagnosis: in a rodent model, acute and chronic hyponatremia reduced hippocampal BDNF expression, with the greatest reduction observed when hyponatremia was combined with seizures and accompanied by marked ultrastructural hippocampal injury (26), supporting the view that circulating and central BDNF may track metabolic and seizure-related physiological state rather than serving as a dedicated marker of cognitive impairment.

Framing BDNF within a neuroimmune rather than a purely neurotrophic model may help reconcile some of this heterogeneity, even if it cannot fully resolve it. In the two studies that measured BDNF alongside inflammatory or oxidative stress markers rather than in isolation, BDNF’s relationship to those co-measured markers was often stronger than its relationship to cognition itself. Sengupta et al. (9) found that, after age adjustment, BDNF did not differ significantly across intellectual disability severity strata, whereas oxidative stress markers measured in the same cohort (reactive oxygen species, lipid peroxidation, superoxide dismutase activity) showed significant, severity-graded differences; and Ng et al. (15) reported a significant association between BDNF and high-sensitivity CRP within their CP cohort specifically. This pattern supports the idea that circulating BDNF reflects a broader neuroimmune and metabolic state rather than a dedicated readout of synaptic or cognitive status, and suggests that future work may find more traction in modelling BDNF jointly with inflammatory and oxidative stress biomarkers, as in Tarani et al. (8), rather than treating it as a stand-alone cognitive biomarker.

Findings from the existing autism meta-analysis provide a useful external comparison. Saghazadeh and Rezaei (10) also reported substantial heterogeneity, alongside a modest, direction-consistent elevation of blood BDNF in ASD relative to controls. The heterogeneity observed here is larger and more erratic, including outright reversals of direction, suggesting that the biomarker instability seen across CP, ID, and DS studies is not merely a general feature of paediatric neurodevelopmental BDNF research but is comparatively more severe in this specific population. A likely reason is that it spans a more etiologically heterogeneous set of conditions (perinatal brain injury in CP; a mixture of genetic, prenatal, and unexplained causes in non-syndromic ID; a single well-defined genetic cause in DS) than autism, which, despite its own genetic heterogeneity, was studied here with more uniform, contemporaneous case–control designs.

Clinically, the present findings argue against using circulating BDNF, on the basis of currently available evidence, as a screening, diagnostic, or severity-stratification tool in children with CP, non-syndromic ID, or DS. Given the null pooled effect, the very high heterogeneity, and the directional inconsistency documented above, a clinician or researcher encountering a single new BDNF value in one of these populations currently has no reliable basis for inferring cognitive severity from it. This should be distinguished from a conclusion that BDNF is biologically irrelevant to these conditions: several of the underlying mechanistic links (TrkB-mediated synaptic plasticity, neuroimmune crosstalk, age-dependent maturation) remain well supported at the basic-science level. Rather, the evidence base linking peripheral BDNF concentration specifically to cognitive severity in these clinical populations is not yet mature or methodologically standardised enough to support translation.

Several limitations of the present review should be considered alongside these conclusions. The number of studies contributing to the main quantitative pool was small (five comparisons from four studies), which limits statistical power to formally test moderators of heterogeneity (e.g., via meta-regression) and precludes a reliable assessment of publication bias using funnel-plot or Egger’s-test approaches, which require a substantially larger number of studies to be informative. Data for one included study (8) were available only as a bar graph rather than in numerical form and were therefore approximated by digitisation; although a sensitivity analysis showed the overall conclusion was not sensitive to this approximation, the underlying uncertainty should be kept in mind. In addition, several included studies (9, 22) lacked an external healthy-control comparison altogether and were analysed qualitatively rather than quantitatively, meaning the main pooled effect size does not draw on the full body of available evidence. Finally, although an *a priori* protocol was prepared before data extraction, the review was not registered in a public registry, which is acknowledged as a further limitation.

Future primary research in this area would benefit from several specific design features that the present synthesis suggests are currently lacking in combination: age-matched healthy control groups (rather than adult or clinically distinct comparators); a single, standardized, well-validated BDNF assay platform used consistently across sites and studies to permit direct comparison and future meta-analytic pooling without unit-conversion or platform-heterogeneity concerns; joint measurement of BDNF alongside inflammatory and oxidative stress markers within the same cohort, consistent with a neuroimmune rather than purely neurotrophic model; and longitudinal, within-subject designs capable of testing whether change in BDNF over time, rather than a single cross-sectional concentration, tracks cognitive trajectory or responds to rehabilitative intervention, an approach only tentatively explored to date (24, 25) and never in the context of a controlled, cognition-focused outcome.

## 5 Conclusion

This systematic review and meta-analysis addressed two related questions in children and adolescents with cerebral palsy, non-syndromic intellectual disability, or Down syndrome: whether circulating BDNF concentration differs between affected and unaffected individuals, and whether it tracks the severity of cognitive impairment within affected populations. Neither question is currently answered by clinically reliable evidence. The pooled case–control effect size across five eligible comparisons was small and statistically non-significant, and was accompanied by very high, largely unexplained heterogeneity that persisted even when the analysis was restricted to a single diagnostic subgroup; a separate narrative synthesis of within-group severity-gradient evidence, though limited to three studies with incompatible effect-size metrics, likewise found no reliable BDNF-severity association. This heterogeneity appears to be driven substantially by methodological factors, particularly differing assay platforms and inconsistent age-matching of comparator groups, rather than by genuine differences in underlying biology between diagnostic categories. Based on currently available evidence, circulating BDNF cannot be recommended as a screening, diagnostic, or severity-stratification biomarker in this population. Its candidacy as a neuroimmune biomarker of either case–control status or cognitive impairment severity remains biologically plausible but empirically unproven, and should be re-evaluated once age-matched, methodologically standardised, and ideally longitudinal primary studies that measure BDNF jointly with inflammatory and oxidative stress markers become available.

## Data Availability

The original contributions presented in the study are included in the article/Supplementary material, further inquiries can be directed to the corresponding author.
