# Supplementary material for: Circulating BDNF in pediatric neurodevelopmental disorders: a meta-analysis of case–control differences and cognitive severity
Source: Front Neurol. 2026 Sep 23;17:1958266. doi: 10.3389/fneur.2026.1958266 (PMC13642140; doi:10.3389/fneur.2026.1958266)
Supplement: Supplementary file 1 [file Table_1.docx]

**Supplementary Table S1. Risk of bias (QUIPS) for included studies**

Assessment with the Quality In Prognosis Studies (QUIPS) tool across six domains: (1) study participation, (2) study attrition, (3) prognostic factor measurement, (4) outcome measurement, (5) adjustment for other prognostic factors (confounding), and (6) statistical analysis and reporting. Ratings are Low, Moderate, or High risk.

| **Study** | **Participation** | **Attrition** | **Prognostic factor** | **Outcome measurement** | **Confounding** | **Statistical analysis** | **Overall** |
| --- | --- | --- | --- | --- | --- | --- | --- |
| Esnafoglu & Adıgüzel (2021) | Moderate | Low | Low | Low | High | Low | Moderate |
| Nelson et al. (2001) | Moderate | Moderate | High | High | Moderate | Moderate | High |
| Nelson et al. (2006) | Moderate | Low | High | High | Moderate | Moderate | High |
| Miyazaki et al. (2004) | High | Low | Moderate | High | High | Moderate | High |
| Tarani et al. (2020) | Moderate | Low | Moderate | High | Moderate | Moderate | High |
| Gamayani et al. (2015) | Low | Low | Moderate | Low | High | Low | Moderate |
| Sengupta et al. (2019) | Moderate | Low | Moderate | Low | Moderate | Low | Moderate |

Assessment. All three authors (BY, BCT, NE) assessed risk of bias together, with domain and overall ratings agreed by consensus. For Tarani et al. (2020), BDNF concentrations were reported only as a bar graph and were approximated by digitization, so this study was likewise retained only for sensitivity analysis.
